# Impact of prone position on dead-space fraction in COVID-19 related acute respiratory distress syndrome

**DOI:** 10.1186/s12890-024-02845-w

**Published:** 2024-01-05

**Authors:** Guillaume Théry, Astrée Scemama, Elvire Roblin, Morgan Caplan, Bruno Mourvillier, Antoine Goury

**Affiliations:** 1 Intensive Care Unit, Reims Hospital University, Reims, France; 2 Department of Biostatistics and Epidemiology, Gustave Roussy, Paris-Saclay University, Villejuif, France

**Keywords:** ARDS, COVID-19, Prone position, Dead-space fraction, EtCO2

## Abstract

**Introduction:**

COVID-19 Related Acute Respiratory Syndrome (C-ARDS) is characterized by a mismatch between respiratory mechanics and hypoxemia, suggesting increased dead-space fraction (DSF). Prone position is a cornerstone treatment of ARDS under invasive mechanical ventilation reducing mortality. We sought to investigate the impact of prone position on DSF in C-ARDS in a cohort of patients receiving invasive mechanical ventilation.

**Methods:**

we retrospectively analysed data from 85 invasively mechanically ventilated patients with C-ARDS in supine and in prone positions, hospitalized in Intensive Care Unit (Reims University Hospital), between November, 1st 2020 and November, 1st 2022. DSF was estimated via 3 formulas usable at patients’ bedside, based on partial pressure of carbon dioxide (PaCO2) and end-tidal carbon dioxide (EtCO2).

**Results:**

there was no difference of DSF between supine and prone position, using the 3 formulas. According to Enghoff, Frankenfield and Gattinoni equations, DSF in supine vs. prone position was in median respectively [IQR]: 0.29 [0.13–0.45] vs. 0.31 [0.19–0.51] (*p* = 0.37), 0.5 [0.48–0.52] vs. 0.51 [0.49–0.53] (*p* = 0.43), and 0.71 [0.55–0.87] vs. 0.69 [0.57–0.81], (*p* = 0.32).

**Conclusion:**

prone position did not change DSF in C-ARDS.

## Introduction

Severe Acute Respiratory Syndrome Coronavirus 2 (SARS-CoV-2) was identified in December 2019 as the cause of coronavirus disease 2019 (COVID-19). Some patients may develop a more severe disease, resulting in acute respiratory distress syndrome (ARDS) when it meets ARDS criteria.

COVID-19 related ARDS (C-ARDS) is characterized by preserved initial lung compliance and severe hypoxemia not fully explained by alveolar lesions [1].

Physiologic Dead-Space Fraction (DSF) is an important index of overall lung function that is strongly associated with more severe disease and lower survival in invasively ventilated patients with ARDS [2]. Increased DSF is a feature of the early phase of ARDS. Despite its diagnostic and prognostic importance, physiologic DSF is not included in the current ARDS definition or severity classification, nor as a treatment target. Measurement of DSF by electrical impedance tomography (EIT) or collection of expired gas into a reservoir bag is hardly feasible at patient’s bedside. Based on Riley’s model of gas exchange [3], PaCO2 depends on the amount of non-aerated lung and on the tension of CO2 in the mixed venous blood. DSF has therefore been estimated by different authors such as Bohr with Enghoff modification, Frankenfield, and Gattinoni [4–6].

In parallel, prone position is a cornerstone treatment of ARDS under invasive mechanical ventilation reducing mortality. A pilot study based on EIT suggested DSF reduction in patients with C-ARDS turned prone, but with a small sample size [7]. Hence, we sought to investigate the impact of prone position on DSF in C-ARDS, but based on bedside measurements.

## Methods

We retrospectively included patients admitted to the ICU who fulfilled inclusion criteria: ≥ 18 years old, admission to ICU for C-ARDS confirmed on real-time reverse transcriptase-polymerase chain reaction (RT-PCR). All patients were under protective mechanical ventilation and met criteria for prone positioning. End-tidal CO2 was measured by a linear capgnography equipment (Microstream™ Advance, Philips). Patients were excluded if they had non-confirmed SARS-CoV-2 infection, no data at baseline or at hospital discharge, or who were admitted to an ICU for other reasons. Patients who required ECMO support were excluded from the main analysis to avoid potential bias in blood gases analysis and pulmonary mechanics.

DSF estimation was made by three formulas: Bohr equation with Enghoff modification: (PaCO2-PEtCO2)/PaCO2, Frankenfield equation: 0.32 + 0.0106*(PaCO2 - ETCO2) + 0.003*(RR) + 0.0015*(age), and Gattinoni equation: PEtCO2/PaCO2 [4–6].

Ventilatory parameters were collected in supine position and during the first prone position session for each patient.

Continuous variables are expressed as median (interquartile range) and were compared using the Wilcoxon test for paired samples. Categorial variables are expressed as n (%) and were compared using the Fisher exact test. A *p*-value < 0.05 was considered statistically significant. All analyses were performed using SAS version 9.4 (SAS Institute Inc., Cary, NC, USA). In agreement with French law and the Commission Nationale de l’Informatique et des Libertés (CNIL) MR-004, written informed consent was not needed for this observational study, but patients and/or their relatives were informed of the use of their data. All methods were performed in accordance with the relevant guidelines and regulations [8].

## Results

Between November 2020 and November 2022, 251 patients with C-ARDS were treated at our centre, of whom 85 who underwent prone position were included (29% women; median age, 62 [49–75] years) (Table 1). Body mass index was 32 (25–39) kg/m2, Simplified Acute Physiology Score II was 41 (25–57), 60% had hypertension, 39% diabetes and 19% received at least one dose of SARS-CoV-2 vaccination. Median number of prone position sessions was 5 [1–9] with a median duration of 18 h (16–20). Median PaO2/FiO2 ratio was 101 (59–143) mmHg. 14% patients had a documented pulmonary embolism and 61% patients had more than 50% pulmonary infiltrates on CT-scan. They were all treated by dexamethasone. All patients received mechanical ventilation through an endotracheal tube under analgosedation (median RASS score − 4), neuromuscular blockade and prone positioning. Norepinephrine was given to 50 (59%) patients. Gas parameters analysis and DSF estimation were made just before prone session and at the end of prone session.

**Table 1:** Baseline characteristics of the study population and events during follow-up

	n (%), [IQR]
**Patients included**	85
**Baseline characteristics**	
Age, years	62 [49–75]
Female sex	25 (29)
SAPS II	41 [25–57]
BMI, kg/m2	32 [25–39]
Hypertension	51 (60)
Diabetes	33 (39)
Cardiomyopathy	23 (27)
Chronic kidney failure	17 (20)
Active Smoking	13 (15)
COPD	14 (16)
Chronic respiratory insufficiency	18 (21)
Sars-CoV-2 vaccination	16 (19)
**Tomodensitometry findings**	
Percentage of pulmonary infiltrate	
0–25%	6 (7)
26–50%	27 (32)
51–75%	30 (35)
> 75%	22 (26)
Pulmonary embolism	12 (14)
**Treatments before invasive mechanical ventilation**	
High-flow nasal oxygen	28 (33)
Non-invasive ventilation	55 (65)
Dexamethasone	85 (100)
Tocilizumab	6 (7)
Nirmatrelvir/ritonavir	1 (1)
Tixagevimab/cilgavimab	2 (2)
Remdesivir	2 (2)
**Intensive care unit**	
Neuromuscular agent blockade	85 (100)
Inhaled NO	24 (28)
Renal replacement therapy	20 (24)
VV-ECMO*	5 (6)
Norepinehrine	50 (59)
Duration of prone position, h	18 [16–20]
Prone position sessions	5 [1–9]
Duration of invasive mechanical ventilation, d	27 [7–47]
Length of ICU stay, d	30 [9–51]
Day 90 mortality	37 (44)
Day 90 ventilatory-free days alive	34 (29)

Abbreviations: BMI, body mass index; COPD, chronic obstructive pulmonary disease; ICU, intensive care unit; IQR, interquartile range; NO, nitric oxide; SAPS, simplified acute physiology score; SARS-CoV-2, severe acute respiratory syndrome coronavirus 2; VV-ECMO, veno-venous extracorporeal membrane oxygenation

*the 5 patients were proned before VV-ECMO initiation

Median PaO2/FiO2 ratio was 101 [59–143] mmHg in supine position vs. 186 [180–266] mmHg in prone position (*p* < 0.001). Hemodynamic and ventilatory parameters are available in Table 2.

**Table 2:** Ventilatory parameters

	**Supine position** **median [IQR]**	**Prone position** **median [IQR]**	**p-value**		
Heart rate, bpm	82 [62–102]	75 [58–92]	< 0,1		
MAP, mmHg	83 [63–103]	80 [69–91]	0,91		
FiO2, %	86 [72–100]	57 [39–75]	< 0,01		
PaO2, mmHg	85 [54–116]	96 [69–123]	< 0,01		
PaCO2, mmHg	43 [33–53]	44 [37–51]	0,076		
PaO2/FiO2 ratio	101 [59–143]	186 [180–266]	< 0,001		
SaO2, %	93 [88–98]	95 [93–97]	< 0,001		
Tidal Volume, mL	399 [341–457]	395 [336–454]	0,46		
Respiratory rate, /min	25 [21–29]	25 [22–28]	0,052		
PEEP, cmH2O	9 [7–11]	10 [8–12]	0,54		
PaCO2-EtCO2	13 [4–22]	14 [8–20]	0,34		
Dead-Space Fraction					
Enghoff	0,29 [0,14 − 0,44]	0,31 [0,19 − 0,43]	0,37		
Frankenfield	0,501 [0,475-0,527]	0,505 [0,478-0,32]	0,43		
Gattinoni	0,70 [0,54 − 0,86]	0,68 [0,56 − 0,8]	0,32		

Abbreviations: EtCO2, end-tidal carbon dioxide; FiO2, fraction of inspired oxygen; IQR, interquartile range; MAP, mean arterial pressure; PaCO2, partial pressure of carbon dioxide; PaO2, partial pressure of oxygen dioxide; PEEP, positive end-expiratory pressure; SaO2, arterial oxygen saturation

**Fig. 1:**
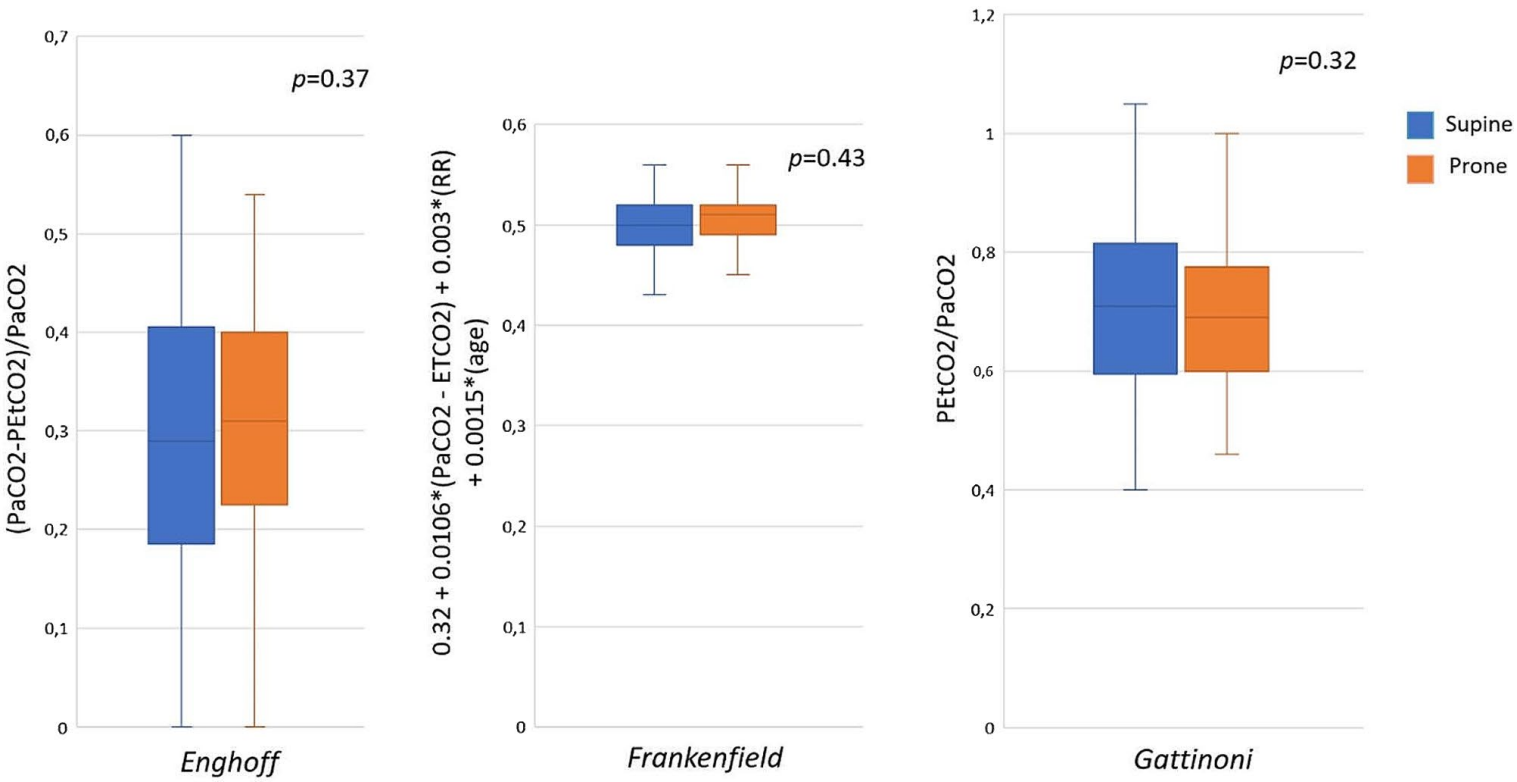
Comparison of dead-space fraction surrogates between supine and prone position. Abbreviations: PEtCO2, end-tidal carbon dioxide pressure; PaCO2, partial pressure of carbon dioxide; RR, respiratory ratio

At the end of prone position sessions, there were no changes of DSF, regardless of the calculation method (Fig. 1). According to Enghoff, Frankenfield and Gattinoni equations, DSF estimation pre- vs. post-prone position were respectively: 0, 29 (0.13–0.45) vs. 0.31 (0.19–0.51) (*p* = 0.37), 0.5 (0.48–0.52) vs. 0.51 (0.49–0.53) (*p* = 0.43), and 0.71 (0.55–0.87) vs. 0.69 (0.57–0.81), (*p* = 0.32).

## Discussion

In this retrospective analysis of 85 intubated C-ARDS patients who performed at least one prone position session, we did not find any impact of prone position on DSF.

Recently, in a C-ARDS population, *Sharp et al.* retrospectively showed an increase in DSF in their non-surviving patients, with no impact of prone position on DSF, and an increase of PaO2/FiO2 ratio [9]. Considering that there is a poor recruitability in COVID-19 patients [10], authors suggest that there might be a decrease in shunt in prone position, with no impact in lung parenchyma, improving V/Q mismatch. Depression of cardiac output is a well-described mechanism of shunt reduction leading to an improvement in V/Q mismatch [11]. Although our results are keeping with *Sharp et al.*’s, explanations about improvement of oxygenation are just hypothesis and could not be proven in our study due to the lack of measurements of lung perfusion and cardiac output.

Interestingly, we can underline that all results were concordant between each 3 formulas. We must specify that we did not compare formulas between them.

Gattinoni’s formula is a good surrogate for both DSF and oxygenation dysfunction in patients with ARDS. Kallet et al. showed in a study including 561 patients with C-ARDS that decreasing EtCO2/PaCO2 in early ARDS was associated with increasing Vd/Vt and oxygenation dysfunction, illness severity scores, and mortality. Moreover, EtCO2/PaCO2 was independently associated with mortality risk after adjustment [12].

Unlike Gattinoni’s formula, Frankenfield and Enghoff equation results are proportional to Vd/Vt.

The fact that C-ARDS is not only a pulmonary disease but also a vascular pathology with capillary thrombosis, might explain the absence of pronation impact on DSF; specific perfusion impairment found in C-ARDS may not be directly changed between supine and prone position. These results are not generalizable to other ARDS.

Unlike our results, van Meen et al., found in a post-hoc analysis including patients with ARDS [13], that prone position induced changes in PaO2/FiO2 ratio, DSF estimated by Enghoff formula, and respiratory system driving pressure with an association with mortality. This difference may be explained by the small sample of the population included and the fact that C-ARDS physiopathology is different from other ARDS, in terms of endothelial involvement and lung recruitability [14].

Another potential explanation is that we measured DSF parameters only at the end of the first prone position session and did not follow its evolution over time, either by taking into account the number of prone position sessions per patient or by measuring DSF during each of them.

Our study had several important limitations. First, this study has a small sample size and was conducted retrospectively at a single-center. Then, respiratory mechanics data are lacking and multiple gas exchanges evaluation during prone position could have been of interest. Also, even if EtCO2 is easily accessible at patient’s bedside, we did not compare our results to other DSF estimation tools, as EIT. In fact, the use of EIT could have been helpful to understand the regional distribution of ventilation/perfusion matching which may not be reflected by the overall dead space fraction estimated by the three equations applied in our study. Zarantonello et al. used EIT in 30 ventilated patients with C-ARDS; prone position overall produced an early increase in ventilation-perfusion matching and dorsal ventilation, while it did not significantly affect ventilation and perfusion homogeneity [15]. Moreover, further statistical analyses were not deemed necessary as the aim of the study was to compare DSF between prone and supine position but other tests might help assessing DSF equations among them. Finally, future studies are warranted to confirm our findings.

In conclusion, in C-ARDS, prone position did not change DSF estimated by 3 formulas, usable at beside patients.

## Data Availability

All data generated or analysed during this study are included in this published article.
